# Therapy-Related Acute Promyelocytic Leukemia Developed During Pembrolizumab Therapy

**DOI:** 10.7759/cureus.37919

**Published:** 2023-04-21

**Authors:** Cecily E Ober, Charles E Jacocks, Michael B Osswald

**Affiliations:** 1 Internal Medicine, San Antonio Uniformed Services Health Education Consortium, San Antonio, USA; 2 Hematology and Oncology, San Antonio Uniformed Services Health Education Consortium, San Antonio, USA

**Keywords:** therapy-related acute promyelocytic leukemia, acute myeloid leukemia, immune-checkpoint inhibitors, acute promyelocytic leukemia, pembrolizumab

## Abstract

A 75-year-old male was diagnosed with carcinoma in-situ of the bladder. He failed standard therapy and was started on pembrolizumab to prevent the need for cystectomy. His malignancy recurred, and he was treated with intravesical valrubicin and gemcitabine/docetaxel. Three years after starting pembrolizumab, he developed severe neutropenia and thrombocytopenia. He was treated for suspected auto-immune cytopenias but was later found to have acute promyelocytic leukemia on peripheral blood smear and cytometry. He was hospitalized, treated with all-trans retinoic acid and arsenic trioxide, and is currently in molecular remission.

This case describes therapy-related acute promyelocytic leukemia (t-APL) diagnosed while on pembrolizumab. Pembrolizumab is an immune checkpoint inhibitor that exhibits anti-tumor effects. Development of hematologic malignancies after immune checkpoint inhibitor therapy is rare. The definitive etiology of our patient's t-APL is uncertain; however, it is more likely that he developed de novo acute promyelocytic leukemia (APL), which was suppressed by pembrolizumab and later revealed when pembrolizumab was discontinued.

## Introduction

Acute promyelocytic leukemia (APL) is a rare type of acute myeloid leukemia (AML) that arises from a chromosomal translocation and subsequent fusion of promyelocytic leukemia (PML) and retinoic acid receptor alpha (RAR) genes from chromosome 15 and 17 [1]. The resulting fusion gene, PML-RARα, inhibits cells from differentiating beyond the promyelocytic stage [1]. PML-RARα is specifically targeted by all-trans retinoic acid (ATRA). APL can be fatal due to aggressive coagulopathies and differentiation syndrome during initial onset. However, when patients are quickly started on ATRA and arsenic trioxide therapy with reversal of coagulopathies, long-term survival rates are around 95% in low, intermediate, and high-risk patients [1-2].

Therapy-related APL (t-APL) accounts for up to 12-14% of APL cases [2-3]. T-APL tends to occur less than three years after treatment of the primary malignancy which are most commonly breast, hematological, or genitourinary malignancies [3-4]. It is also a known complication of systemic topoisomerase II inhibitors, specifically epirubicin, etoposide, and mitoxantrone [4]. Studies have shown that t-APL and de novo APL are cytogenetically similar with the same chromosome 15 and 17 translocation (t(15;17)) [2-4] and have morphologically and phenotypically identical promyelocytes [2]. Therefore, in order to be considered t-APL, a t(15;17) mutation must be present, the onset occurs after chemotherapy or radiotherapy, and the patient cannot have a previous myeloproliferative neoplasm [3]. Patients tend to have no preleukemic phase, and in one-third of patients, t-APL is identified incidentally on routine follow-up [3-4]. Patients commonly present with mucocutaneous bleeding, and approximately 80% of patients present with clinical disseminated intravascular coagulation (DIC) [3]. The prognosis of t-APL and de novo APL are comparable with early treatment initiation [2-3].

Here we present a case of pembrolizumab-related acute promyelocytic leukemia.

## Case presentation

A 75-year-old male was diagnosed with non-muscle invasive carcinoma in-situ of the bladder in August 2018. Over the next year, he was treated with and failed two rounds of bacillus Calmette-Guérin (BCG) therapy. In October 2019, he started pembrolizumab treatment to prevent the need for a cystectomy. Over the next few years of treatment, he subsequently developed multiple autoimmune side effects from pembrolizumab, including adrenal insufficiency, hypophysitis, hypothyroidism, hepatitis, and arthritis, which were all successfully treated with either temporarily discontinuing pembrolizumab or hormonal replacement. Due to the recurrence of his cancer, throughout 2021, he received intravesical valrubicin and intravesical gemcitabine/docetaxel while on pembrolizumab. His bladder cancer was successfully treated with this regimen, and he continued on pembrolizumab to prevent recurrence.

**Figure 1:**
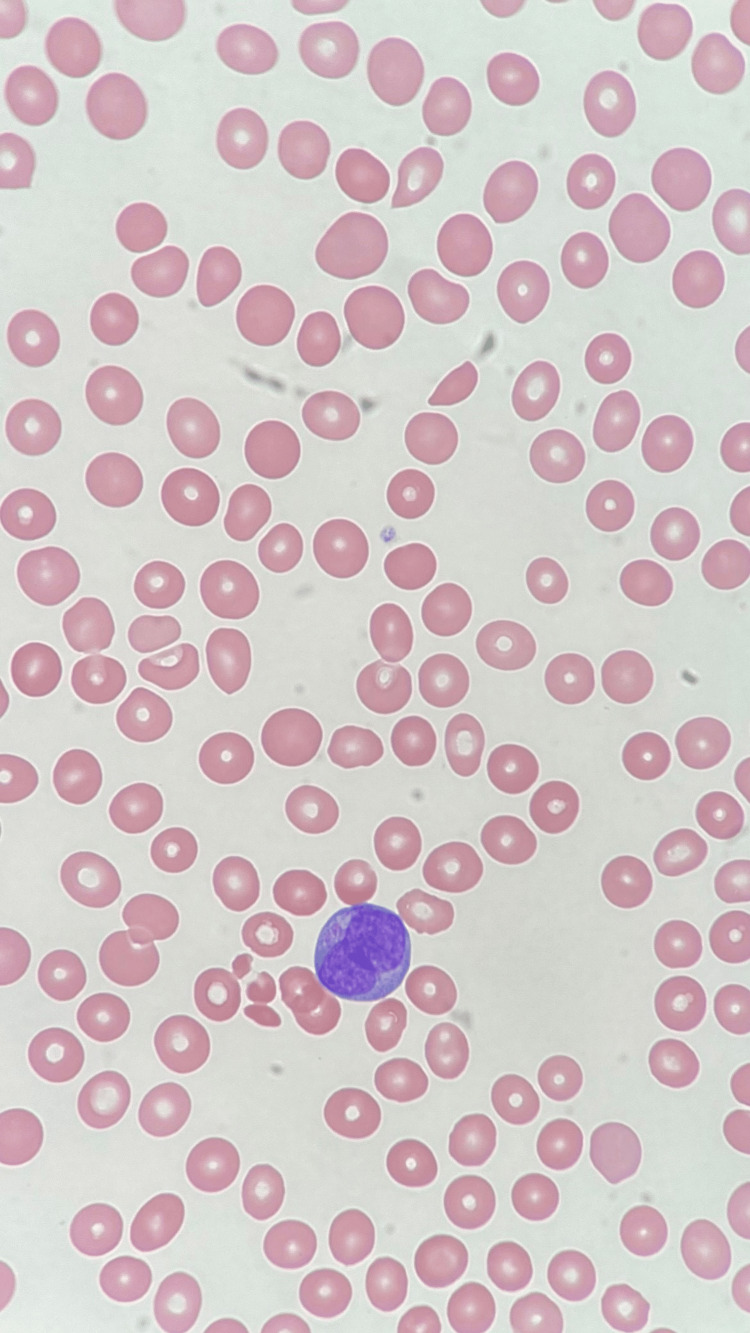
Single promyelocyte with classic sliding plate morphology with evidence of schistocytes consistent with DIC on presentation DIC: Disseminated Intravascular Coagulation

On routine labs in the fall of 2022, he was found to have severe neutropenia (ANC 0.34 x 10^6/uL) and moderate thrombocytopenia (PLT 58 10^3/uL), which were thought to be autoimmune cytopenias, a known side effect of pembrolizumab. Peripheral blood smear did not demonstrate any dysplasia or immaturity. Pembrolizumab was stopped, and he was given steroids, a dose of pegfilgrastim, and two doses of intravenous immune globulin (IVIG). He developed an appropriate leukocytosis to this therapy; however, his platelet count did not improve. When the leukocytosis did not spontaneously resolve after two weeks, he underwent further testing, and peripheral blood smear showed the presence of promyelocytes (Figure 1). 

Peripheral blood flow cytometry showed APL with a t(15:17) translocation on fluorescence in situ hybridization (FISH) analysis. Upon diagnosis, he was quickly admitted to the hospital for treatment of APL and was found to be in moderate DIC on presentation with severe thrombocytopenia (Table 1). 

**Table 1:** Complete blood count and coagulation data on the day of t-APL diagnosis CBC - complete blood count; WBC - white blood cells; Segs - segmental neutrophils; Lymph - lymphocytes; Mono - monocytes; Eos - eosinophils; Baso- basophils; Promyelo - promyelocytes; RBC - red blood cells; Hgb - hemoglobin; Hct - hematocrit; MCV - mean corpuscular volume; PLT - platelet; PT - prothrombin time; PTT - partial thromboplastin time.

CBC		Normal range
WBC	16.25 x10^3^/uL	(3.60-10.60)
Segs	8.00%	(41.0-73.0)
Lymph	18.00%	(18.0-46.0)
Mono	3.00%	(0.0-10.0)
Eos	2.00%	(0.0-6.0)
Baso	0.00%	(0.0-2.0)
Promyelo	1.00%	0
RBC	2.73 x10^6^/uL	(4.2-6.0)
Hgb	9.0 g/dL	(13.5-18.0)
Hct	24.30%	(40.0-54.0)
MCV	98.2 fL	(80.0-100.0)
PLT	10 x10^3^/uL	(150-450)
Coagulation		Normal range
PT	16.3 seconds	(12.3-14.6)
PTT	28.0 seconds	(24.2-33.2)
Fibrinogen	80.0 mg/dL	(213-462)
D-Dimer	16.33 mcg/mL	(0.00-0.49)
Thrombin time	22.8 seconds	(14.4-18.1)
Haptoglobin	<10 mg/dL	(30-200)

He showed a therapeutic response to ATRA and arsenic trioxide therapy. During hospitalization, he was treated with steroids for his autoimmune adrenal insufficiency and the prevention of differentiation syndrome. His DIC resolved. He was discharged after a few weeks following induction therapy with ATRA and arsenic trioxide, where he was continued on consolidation therapy as an outpatient. He is currently in complete molecular remission.

## Discussion

Pembrolizumab is an immune checkpoint inhibitor, specifically, an immunoglobulin G4 (IgG4) monoclonal antibody against the checkpoint programmed cell death-1 (PD-1) [5]. PD-1 is an immune response checkpoint that is expressed by activated T cells. Because ligands of PD-1 are found on the surface of tumor cells and macrophages, binding of PD1 to its ligand (PD-L1) allows tumor cells to grow unchecked by the immune system [5]. By inhibiting the receptor/ligand binding, pembrolizumab exhibits anti-tumor effects giving the immune system the ability to act against the tumor cells [5]. It is currently approved to treat multiple cancer types, including melanoma, bladder cancer, and lung cancer [5-6].

Pembrolizumab tends to cause fatigue, diarrhea, nausea, rash, and arthralgias [5]. Because of its physiologic mechanism of action, immune-related adverse events (irAE) can occur. Endocrine irAEs such as hypothyroidism/hyperthyroidism and hypophysitis are the most frequent, but irAEs for every organ system have been reported [5,7]. Hematologic toxicities include anemia and thrombocytopenia, and although rare, neutropenia has been reported [7]. Our patient developed autoimmune hypothyroidism, hepatitis, arthralgias, and what was initially thought to be autoimmune neutropenia and thrombocytopenia.

It is difficult to specifically identify the etiology of our patient's t-APL. It presented approximately 18 months after receiving intravesical valrubicin, 10 months after intravesical gemcitabine/docetaxel, and three years after initiation of pembrolizumab. Topoisomerase II inhibitors are known causes of t-APL [4]. However, research has shown that intravesical valrubicin is absorbed systemically but with low plasma levels [8], and intravesical docetaxel is not absorbed systemically [9], so these drugs are less likely to be the cause of his t-APL. It is possible that our patient developed de novo APL unrelated to chemotherapy which was suppressed while he was on pembrolizumab. Thus, when he was treated for presumed autoimmune neutropenia and thrombocytopenia with steroids and pegfilgrastim, with subsequent discontinuation of pembrolizumab, he developed this APL presentation.

Hematologic malignancies following treatment with immune checkpoint inhibitors have rarely been reported [10], although no association between iRAEs and hematologic malignancies have yet to be established [1,10]. There is only one other reported case of pembrolizumab-related APL in which a patient developed t-APL after treatment with pembrolizumab and docetaxel therapy for lung carcinoma and epirubicin for hepatocellular carcinoma [1]. The patient was diagnosed with APL approximately eight months after completing systemic docetaxel and approximately two months after stopping pembrolizumab. Docetaxel was thought to be the primary cause of the t-APL; furthermore, the patient developed severe thrombocytopenia after discontinuation of pembrolizumab, which could represent pembrolizumab suppressing t-APL and subsequent worsening of his t-APL after discontinuation of pembrolizumab [1]. Researchers have hypothesized that hyperprogression of secondary lymphoproliferative diseases through the PD-1 blockade may occur after immunotherapy [10]. Other research has shown that a blockade of PD-1/PD-L1 could be a treatment option for AML [1]. Research focused on the use of immune checkpoint inhibitors such as pembrolizumab alone or in combination with other agents in treatment for AML has not yet shown clinical significance [1].

## Conclusions

In summary, this case describes therapy-related APL diagnosed while on pembrolizumab. Development of hematologic malignancies after immune checkpoint inhibitor therapy is a rare occurrence. The definitive etiology of our patient's t-APL is uncertain; however, it is more likely that he developed de novo APL, which was suppressed by pembrolizumab and later revealed when pembrolizumab was discontinued. Further research is needed to evaluate the efficacy of immune checkpoint inhibitors in APL and AML treatment.
